# Factors affecting pharmacists’ recommendation of complementary medicines – a qualitative pilot study of Australian pharmacists

**DOI:** 10.1186/1472-6882-12-183

**Published:** 2012-10-10

**Authors:** Sarah E Culverhouse, Hans Wohlmuth

**Affiliations:** 1 School of Health and Human Sciences, Southern Cross University, PO Box 157, Lismore NSW 2480, Australia; 2 Southern Cross Plant Science, Southern Cross University, PO Box 157, Lismore NSW 2480, Australia

**Keywords:** Pharmacy and complementary medicine, Pharmacists’ attitude towards complementary medicine, Pharmacy practice, Companion selling, Qualitative study

## Abstract

**Background:**

Complementary medicines (CMs) are widely used by the Australian public, and pharmacies are major suppliers of these medicines. The integration of CMs into pharmacy practice is well documented, but the behaviours of pharmacists in recommending CMs to customers are less well studied. This study reports on factors that influence whether or not pharmacists in Australia recommend CMs to their customers.

**Methods:**

Data were collected from semi-structured interviews with twelve practicing pharmacists based in Brisbane, Australia. The qualitative data were analysed by thematic analysis.

**Results:**

The primary driver of the recommendation of CMs was a desire to provide a health benefit to the customer. Other important drivers were an awareness of evidence of efficacy, customer feedback and pharmacy protocols to recommend a CM alongside a particular pharmaceutical medication. The primary barrier to the recommendation of CMs was safety concerns around patients on multiple medications or with complex health issues. Also, a lack of knowledge of CMs, a perceived lack of evidence or a lack of time to counsel patients were identified as barriers. There was a desire to see a greater integration of CM into formal pharmacy education. Additionally, the provision of good quality educational materials was seen as important to allow pharmacists to assess levels of evidence for CMs and educate them on their safe and appropriate use.

**Conclusions:**

Pharmacists who frequently recommend CMs identify many potential benefits for patients and see it as an important part of providing a ‘healthcare solution’. To encourage the informed use of CMs in pharmacy there is a need for the development of accessible, quality resources on CMs. In addition, incorporation of CM education into pharmacy curricula would better prepare graduate pharmacists for community practice. Ultimately, such moves would contribute to the safe and effective use of CMs to the benefit of consumers.

## Background

The popularity and use of complementary medicines (CMs) is increasing in Australia and many other developed countries. A 2004 study in South Australia found 52.2% of those surveyed used some form of CM [1], and a 2007 national survey found that 68.9% of Australians used at least one form of CM in the previous year [2]. As community pharmacy is a major provider of CM products, it is also where many consumers seek advice about their use. There has been limited research, however, into the integration of CM into pharmacy practice and pharmacists’ ability to meet their patients’ needs for CM advice [3,4]. According to a recent survey of pharmacy customers in Australia, 87% expect the pharmacist to be able to recommend efficacious CMs and 92% expect them to provide information relating to the safety of the products [5]. Pharmacists generally agree that they should provide this information, but many feel they have insufficient knowledge or education [4,6-12]. They do however, view CMs as useful – a survey of 1500 Australian pharmacists found 77% agreed that CMs are a useful supplement to conventional medicine [6], and a recent survey of rural Australian community pharmacists reported that 94% believed they should regularly ask consumers if they are using CMs [11]. The existing research investigating the recommendation of CMs by pharmacists has identified a number of factors that influence this behaviour. These include an understanding of the benefits of CMs for maintenance of general health, prevention of disease and treatment of minor conditions [7,8,13,14], patient demand [7], personal usage of CM [10,11], knowledge of a particular CM product and its proven mechanism of action [6,7], and the profile of the CM company [7]. Whilst profit is not stated as an important motive [6,7,14,15], most pharmacists believe they should sell CMs [7], and that CMs can enhance the image of the pharmacy, increase annual sales and be an important part of the financial business of retail pharmacy [6,16]. The major barrier to the recommendation of CMs by pharmacists is reported to be a lack of evidence [4,6,11,12,17-20]. Other barriers include lack of training, lack of accurate and easily accessible information, lack of subsidies or reimbursement, legal concerns, time constraints, and the fact that other staff in a pharmacy may recommend these products [4,14,16,18,20].

This study aimed to explore, through thematic analysis of semi-structured interviews, what influences a pharmacist to recommend or not recommend CMs to a customer in a community pharmacy setting. A better understanding of these issues may assist in determining the best ways to facilitate better integration of CM into community pharmacy practice, which in turn may benefit both pharmacists and consumers.

## Methods

In the first stage of recruitment, all pharmacists working in a pharmacy in the greater Brisbane area with a current account with CM company Blackmores Ltd. (comprising approximately 90% of Australian pharmacies) were eligible. Potential respondents were contacted by mail and follow up phone call. A second round of recruitment was initiated from recommendations of initial respondents (snowball sampling) and from contacting key personnel in the industry including store managers and Pharmacy Guild staff. Further recruitment was conducted by doorknocking local pharmacies. In these further stages of recruitment, all pharmacists were eligible, including those without a Blackmores account. The 12 recruited pharmacists were assigned to one of three groups based on their responses to a questionnaire provided in the initial information kit. Group assignment was as follows: Group 1 recommended CMs less than 20 times per week, Group 2 recommended CMs 20 to 50 times per week, and Group 3 recommended CMs more than 50 times per week. Where a pharmacist did not work full-time on the pharmacy floor, assignment was made on a pro-rata basis.

Data were collected by semi-structured interviews in locations convenient to respondents, mostly in a private office in the pharmacy or occasionally in a local coffee shop. The questions posed led the discussion to cover a number of areas of interest, namely factors affecting their recommendation of CMs, family and personal use of CMs, information sources for CMs, patterns of CM recommendation, knowledge of CMs, and pharmacy protocols for the recommendation of CMs. Interviews were recorded and manually transcribed; written notes were made of any relevant casual discussion occurring prior to or after the recorded interview. Respondents were invited to check the accuracy of the transcript to ensure it reflected the reality of the interview as they perceived it and to provide an opportunity to add any further information.

Thematic analysis [21] of the transcribed interviews was carried out by the primary investigator (SEC) in consultation with the other investigator. First the transcripts were read and initial codes assigned. Further analysis revealed distinct themes relating to the respondents’ background, education, attitudes to CM and CM recommendation patterns. This allowed for codes to be clustered into descriptive categories, each comprising several subcategories. Data were compared within groups and excerpts selected which illustrated the categories. Data were then compared between groups to identify differences and similarities. All coding, analysis, and interpretation was continuously compared and reassessed to ensure validity, until theoretical saturation was reached [21-23].

The study was approved by the Human Research Ethics Committee of Southern Cross University (approval number ECN-10-224), and all respondents provided written, informed consent.

## Results

A total of 12 pharmacists (five female, seven male, aged 25–60) practising in community pharmacies in the greater Brisbane area (Queensland, Australia) were interviewed in April-May 2011. Their tenure in pharmacy ranged from one-and-a-half years to 36 years. They worked in pharmacies of varying size, both independent and banner pharmacies (banner pharmacies are independently owned pharmacies operating under the same marketing banner) (Table 1). All respondents had received their pharmacy training from one of five Australian universities. Two had received some level of training in CM from private education providers, and all but two had attended industry-sponsored CM seminars. A total of seven hours of interviews were recorded and transcribed (median duration 35 min, range 14–81 min).

**Table 1:** Details of respondents and their pharmacies

**Respondent**	**Years since graduation**	**Pharmacy type**^**†**^	Size of pharmacy CM business*
**Group 1** (<20 times/week)			
1.1	10	Banner	Small
1.2	4	Banner	Small
1.3	2	Independent	Small
**Group 2** (20–50 times/week)			
2.1	3	Banner	Large
2.2	32	Independent	Small
2.3	3	Banner	Medium
2.4	7	Banner	Large
2.5	7	Banner	Small
**Group 3** (>50 times/week)			
3.1	36	Independent	Small
3.2	4	Banner	Small
3.3	32	Banner	Large
3.4	14	Banner	Large

Group 1 respondents recommended CMs <20 times per week, Group 2 respondents 20–50 times per week, and Group 3 respondents >50 times per week.

^†^ Banner pharmacies are independently owned but operate under the same marketing banner.

* Based on annual purchases of products from Blackmores Ltd. Small: <$30,000 Medium: $30,000 - $60,000 Large: >$60,000.

### Identified themes

Six major categories emerged as a result of the data analysis, each with several subcategories (Table 2).

**Table 2:** Major categories and sub-categories identified in transcribed interviews

**Major category**	**Sub-categories**
1. Drivers of the recommendation of CMs	Health benefit
	Condition-based recommendation
	Evidence for efficacy
	Customer demand
	Companion selling and pharmacy protocols
	Profile of company sponsoring product
	Cost
	Ethical responsibility
	Holistic care
	Demographics
	Profitability
2. Barriers to the recommendation of CMs	Safety concerns
	Lack of knowledge about safety and efficacy
	Lack of evidence
	Lack of clear patient benefit
	Fad products
	Time constraints
3. Attitude to CMs	Role for CMs in pharmacy
	Responsibility to provide information about CMs
4. Education and resources	University training
	Work experience
	Self-awareness of knowledge level
	Information sources
	Information needs
5. Personal and family use of CMs	Personal use
	Use by family members
6. Relationship with other healthcare professionals	Medical practitioners
	Naturopaths

Thematic analysis of transcripts involved a process of coding and clustering of codes into categories with continuous comparisons and re-assessment to ensure validity.

There was a large degree of inter-group congruence in responses – with two exceptions. One respondent in Group 1 expressed a greater amount of knowledge and acceptance of CM than others in this group, but stated that they did not recommend CMs extensively, as this was being done by others – both by a naturopath in the store and by local doctors, who made a high level of CM recommendations and directed their patients into the pharmacy with CM prescriptions. The other exception was a pharmacist in Group 2, who had extensive training in CM and was passionate about its benefits. This respondent stated that the reason for their medium (rather than high) level of recommendation of CMs was the conservative demographics of the suburb where the pharmacy was located.

### Drivers of the recommendation of CMs

There were a number of subcategories of this category, especially in Groups 2 and 3, reflecting a wider range of reasons for recommending CMs in these groups (Table 2).

#### Group 1 (low level of CM recommendation)

The main subcategories in this group were ‘condition-based recommendation’ and ‘health benefit’ with a lesser emphasis on ‘customer demand’, ‘company profile’ and ‘cost’.

Each Group 1 pharmacist mentioned between one and four specific CMs and related indications they felt comfortable with. They all mentioned that they stick to this narrow range of CMs:

‘B group vitamins to stressed students or professionals. Glucosamine and fish oils to people with osteoarthritis. Fish oils to people with hypertension. They are my safe zones.’

Customer demand was not mentioned as a strong reason to recommend, except for one respondent who said that if a customer asked for something in particular, that would be their primary reason for selling a CM product.

The profile of a company in terms of reputation and quality of products and services also had some impact on which products were recommended:

‘Credibility and availability long term are things…if considering three or four companies that look after us reasonably well, at least in regards to supply, after that criteria (sic) is met I try to provide value for customers.’

Responses to whether cost was an issue for them or their customers varied:

‘I might spend too much time in trying to give value to people. I’ll walk around to find three brands of fish oil and give them the cheapest at the time.’

‘I found where I was working before, people didn’t find the cost an issue.’

#### Group 2 (medium level of CM recommendation)

The most consistent motives for CM recommendation in this group related to health benefits for patients and companion selling a CM product with a pharmaceutical medication. Subcategories were ‘condition-based recommendation’, ‘health benefit’, ‘customer demand’, ‘evidence for efficacy’, ‘companion selling and pharmacy protocols’, ‘profitability’, ‘demographics’, ‘company profile’, ‘ethical responsibility’ and ‘holistic care’. Cost was mentioned as a factor in their decisions of which CM to recommend, but was not seen as a particularly important one.

Each Group 2 pharmacist discussed between four and ten different CMs they recommended for specific conditions, although they mentioned being comfortable with many more products:

‘A lot [of my recommendations] would be in the top twenty, like fish oil, glucosamine, acidophilus, coming to winter season now, things for the immune system, olive leaf, that I would usually recommend.’

Subcategories of ‘health benefit’, ‘customer demand’ and ‘evidence for efficacy’ were linked for pharmacists in this group. They valued clinical evidence, but feedback from customers also gave them more incentive to recommend CMs:

‘…[I’d like to see] the trials that have been done…but also, if the patient takes it and finds it works, you’ve got to go with that… the most important thing is that it works.’

‘…customers and consumers want to have alternative medicines. They’re actively seeking something else apart from orthodox medicine, so I guess you just have to know about them, and as a pharmacist you have to find whether certain things are evidence based.’

Personal experience of benefit was also described as a motivator to recommend CMs:

‘I think another thing that would influence me is if I’ve had personal experience with a product, so for example if I’ve used something that I’ve found very useful,… and if you tell a customer, “Oh, I’ve used this product and this is what happened” they are more receptive to that product.’

All pharmacists in this group described a preference for a CM over a pharmaceutical medicine in some instances, and they felt quite comfortable talking to customers about this:

‘…the one that probably comes to mind most is for restless legs and cramping. I’ll always pick a calcium-magnesium instead of a sedating antihistamine to relax the muscles.’

Two respondents mentioned benefits in relation to insomnia:

‘If they came in with a sleep issue, I’d think about a natural product first…(because of) side effects, addictiveness.’

Companion sales and pharmacy protocols were a major reason to recommend CMs in both Group 2 and 3. All pharmacists in Group 2 described protocols in their workplace to recommend a particular CM with a particular prescription or over-the-counter (OTC) medication, either to ameliorate side effects or to provide an adjunct therapeutic benefit. The most commonly mentioned combinations were probiotics with antibiotics, co-enzyme Q10 with cholesterol-lowering ‘statin’ medications, glucosamine and/or fish oil with various arthritis medications, and CMs for cold and ‘flu with OTC products:

‘We have a system where if I’m dispensing a prescription medicine, lets say a cholesterol-lowering product, we have tags we put in the basket saying, “Your pharmacist recommends you take CoQ10” or things like that, so even if I am busy doing other things, there is some pharmacist intervention to recommend a product…’

Respondents who worked in banner pharmacies described a corporate policy of companion selling:

‘It’s all written down in the [pharmacy group] protocols, so if you work for [pharmacy group] you’re expected to follow this.’

The benefits to patients of recommending CMs along with conventional medicines was clear to respondents in this group:

‘I’m always in favour of adding something, as long as it doesn’t interact with other medicines… Adding something into their medication profile just to make sure they’re getting a little bit of extra help, and… they don’t need to go on another or higher dose cholesterol tablet, when they can start fish oil instead.’

Whilst they were aware that companion selling was potentially profitable for the pharmacy, respondents denied that would be a reason for following a protocol:

‘If there’s an opportunity to companion sell and they don’t need it, I won’t do it.’

Pharmacists were less likely to recommend CMs when they worked in a pharmacy in a lower socioeconomic area. All respondents currently worked in urban or suburban pharmacies; however, those who had previously worked in regional areas described a greater acceptance of CM in the city. One respondent mentioned that the conservative nature of the suburb where their pharmacy was located influenced recommendation patterns and made them less likely to recommend in some instances. Another pharmacist working in an area with a higher Asian population reported:

‘With the higher Asian population we do sell a lot more vitamins, compared to where I have been previously… The Asian population seems to be much more into natural health and vitamins.’

The respondents’ impression of a CM company’s reputation had some impact on their choice of product. They were as a group more comfortable with brands that were well established and had a larger portfolio of products:

‘…it’s to do with their reputation too…they have good products, they have a good range of products. I’d be less likely to trust a company that put out one wonder product and that’s all they have. So larger companies and more reputable companies I do trust and am more receptive to their new products.’

Some pharmacists in this group expressed the opinion that it was their ethical responsibility in some cases to recommend CM in order to provide proper care to their patients. The term ‘holistic’, a term commonly used by complementary medicine practitioners, was used in regard to their recommendations a number of times:

‘…trying to think holistically how you can improve their health outcomes…that’s one of the main driving factors for me.’

‘…my conscience tells me that I really do need to offer that [advice on CM] to those people who are really interested, and also to educate those people who have been to some degree fobbed off [by orthodox approaches to medicine].’

Finally, Group 2 pharmacists were aware that when they recommended a CM, this recommendation was likely to be taken more seriously because of their status as a trusted healthcare professional:

‘I think coming from a pharmacist, if I am recommending a complementary product as opposed to an assistant, it provides more credibility, trusting that product, they will give it a go, will be more compliant to it as well.’

#### Group 3 (high level of CM recommendation)

Pharmacists in Group 3 had similar reasons for recommending CM to those in Group 2, hence the subcategories describing these reasons were the same for both groups. The most common primary motive for recommending a CM was health outcomes, and whilst they were more candid than pharmacists in other groups about potential profitability, they did not describe this as their primary motive for recommendation of CMs, but rather a beneficial spin-off of good customer service:

‘Well, [I recommend CM] because it works and your customer will come back, and you’ve got a happy customer. A happy customer talking to all their friends, saying, “this chemist really knows what they’re talking about” is the best kind of advertising you can get.’

‘…it’s a twofold thing. You’re helping your customer, and it is important to your pharmacy, so I can’t understand why pharmacists wouldn’t want it.’

Like Group 2, condition-based recommendations were frequently mentioned, and the number of CMs mentioned was similar. Recommendations alongside pharmaceutical medicines were also very common. There were protocols in place in all pharmacies to promote this, and this practice was seen as providing considerable health benefits to the customer:

‘I’d use it as a first line too, but I’d say that probably 80-90% would be as a combination… you give them what they’ve asked for, and then you add in something that will actually help them.’

The Group 3 pharmacists agreed that recommending companion products provides a holistic solution, whatever those products might be:

‘…it’s about health outcomes. It’s not about selling products for the sake of selling products. If you’ve got a really bad dermatitis, it’s no good just giving you a tube of cream. We’ve got to give [companion products]. So it’s about a … healthcare solution. Now if I just gave you that tube of ointment, it might clear it up today, but it would be back tomorrow, [but] if we’d sold the three products at once there’s every chance that will never recur, and you can keep it under control. The patient’s happy as Larry, and it will have cost them less as well.’

A preference for a CM over a pharmaceutical was reported by several respondents:

‘…pain medications with tension headaches. You get a lot of that…from computer use… so I get them onto a magnesium supplement…. and the number of people who will come back within three days and say “that’s the first time I’ve never taken Mersyndol in my life and I’m just ecstatic!”’

Like the pharmacists in Groups 1 and 2, Group 3 respondents wanted good quality evidence, and they felt confident in the evidence they had found:

‘…say a medication is depleting something in the system, say your statins and your CoQ10s… everything that’s evidence-based.’

This group had similar opinions to Group 2 about the importance of the profile of the company and the quality of its products:

‘I think you do have a level of safety…a brand like [mentions three brands] are the brands that have a lot to lose, if they bring out dodgy products into the market. A company that has only one item, they have to work a lot harder to convince me to recommend that product.’

‘You’ve got to have a good product. It’s not about selling something just for the sake of selling something. You’re selling a product you believe in.’

### Barriers to the recommendation of CMs

There were more similarities between groups when it came to the reasons given for not recommending CMs. The major barrier for all groups was safety concerns around patients on multiple medications or with complex health issues, and the perceived need for those patients to seek medical advice before adding a CM to their list of medications:

‘[I wouldn’t recommend to] people that are on a lot of things already. I wouldn’t tell them to take it, unless they’ve spoken to the doctor about it as well…’

‘… if they’re on something that has a narrow therapeutic index, like chemo medications, etc., I’d probably recommend them not to take it [CMs]… and the other one is pregnancy, because there’s a lot of CMs that don’t have safety data…’

Group 1 mentioned a lack of knowledge regarding efficacy and safety of a product as a major disincentive:

‘… even if I know it works, if I don’t know if it’s OK with all the other stuff they’re taking, I can’t recommend it.’

Group 2 stressed the importance of evidence, and consistently mentioned that if this is insufficient, they would not recommend CMs:

‘If there was no evidence behind, I wouldn’t actively recommend it.’

They described their position as one where patient benefit outweighed any profit motive, and they were disinclined to recommend a CM if the patient benefit was not clear:

‘I’m very hesitant to put them on something that doesn’t benefit them, that just benefits the business… it’s an ethical stance that every pharmacist should have….’

They generally had a low opinion of what may be described as ‘fad’ products:

‘They want the magic weight loss thing they saw on “Today Tonight” last night, and I think….hang on….what was it? What rubbish….’

All Group 2 respondents said they wouldn’t recommend ‘fad’ products but the majority had these products in their pharmacies and believed the customer had a right to choose them:

‘If there was a fad product and there was a consumer demand for it, of course we have to provide for that consumer demand, but if there was no evidence behind, I wouldn’t actively recommend it.’

Group 3 identified fewer barriers to the recommendation of CMs, but were also uncomfortable with ‘fad’ products and had some ethical concerns about their presence in a pharmacy. This group expressed that they would be likely to talk someone out of buying such a product, if they were asked their opinion:

‘We don’t sell charlatan-type products… let’s have some evidence-based stuff…that sort of thing tends to be bad for the industry as a whole.’

Three out of the four respondents in this group said that a lack of time was an issue:

‘…we’re under pressure with time……if you stop the next person from being served, because you’re having an extra thirty seconds with someone, who should have been a pretty in and out sort of transaction or interaction, that next person becomes annoyed.’

### Attitude to CMs

Attitudes relating to the value of CMs and to the role of the pharmacist in relation to the use and integration of CMs into pharmacy practice were well delineated between groups. All agreed, however, that pharmacists had a responsibility to provide information to customers about any possible safety issues, such as interactions with pharmaceutical medicines and contraindications.

Group 1 felt CMs had a role to play in terms of health outcomes, but were less inclined to embrace a greater integration of CM into pharmacy practice and did not see it as their role to make CM recommendations. Group 2 saw a growing role for CMs in pharmacy, but were divided on whether it was the role of a pharmacist to recommend them. Most saw the provision of CM advice as adding value to what they could offer to facilitate positive health outcomes, and in doing so gain loyalty from their customers. When asked if they thought CM was going to play a larger role in pharmacy in the future, they were clear that they thought it would:

‘Yes, I think so, yes. It’s got a big role to play.’

Group 3 were very clear that there is an important and growing role for CMs in pharmacy. They said their customers expected them to be able to advise on which CM products were effective. They agreed with those in Group 2 who found it an important aspect of the service they provide and saw it as *‘the area…to focus on in pharmacy in the future’*.

### Education and resources

All but one of the respondents stated they did not receive much CM training during their pharmacy degree All said that they had learnt more about CM from their day-to-day work experience than from their university education or any other source. Questions regarding the adequacy of CM topics in the pharmacy curriculum and whether there should be more of it, provoked differing responses.

Group 1 was, overall, not unhappy with their education in terms of CM. They felt it was not comprehensive and did think it may be useful to incorporate a greater CM component to assist with patient queries:

‘It was a good foundation…it would have been useful, I suppose, to do one semester unit on complementary medicines, particularly for people going into community pharmacy.’

One respondent found the desire to learn more was based upon customer demand:

‘People come in and ask you about it all the time, so you can’t exactly not know about it.’

Group 2 was somewhat more enthusiastic about increasing the level of CM education. They saw this as an advantage to their practice, and one respondent commented that pharmacy students do not become aware of potential benefits until they work in a pharmacy:

‘Yes, I do [think CM education should be incorporated]….If they don’t work in a pharmacy, they don’t realise how much it can influence their scripts and doing a service for the customer and delivering holistic health benefits.’

One Group 2 respondent was rather more sceptical about the acceptance of CM by the pharmacy establishment:

‘I think the way pharmacy is, I doubt whether they would [integrate CM]….they’ve been brainwashed to the point that they don’t want to know about that stuff.’

Group 3 respondents were the most enthusiastic about further integration. They saw benefits in patient care but also mentioned professional obligations such as the need to gain Continuing Professional Development/Education (CPD/CPE) points and medication profiling responsibilities (guidelines for medication profiling services have been developed by the Pharmaceutical Society of Australia to assist customers and their healthcare team in the safe and most effective use of medications [24]):

‘There’s a big need for pharmacists to get educated, so they know about interactions and also the drug profiling that’s coming into pharmacy, that we’re all going to be expected to do.’

Information sources used for CMs varied between groups. Those in Group 1 described rarely feeling a need to look something up. If they did, they tended to use traditional sources such as medical journals, pharmacy trade journals and medical websites. One pharmacist would ask the naturopath who worked in their store. They generally described the data as not compelling:

‘It’s very hard to expand the products that you like to recommend based on evidence, because there’s not always that much out there.’

Those in Group 3 used a wider range of resources, including ones dedicated solely to CM, such as herbal medicine and nutrition textbooks and computer databases such as *Hyperhealth*[25]. They also used information provided by companies and more traditional sources such as pharmacy and medical journals, MIMS (http://www.mims.com.au) and the Australian Medicines Handbook (http://www.amh.net.au). They would also ask a naturopath, if one worked in their store. They described a large amount of information as being available:

‘….and a lot more information is coming through to us in pharmacy. There’s quite a few different sources…The [Pharmacy] Guild will put out notices, Pharmacy Daily….so there’s a lot more of that succinct information, you can then delve into more detailed information if required.’

Those in Group 2 also described using a variety of sources, such as MIMS, the Australian Medicines Handbook and information provided by companies, but rarely used herbal or nutrition textbooks.

All respondents described a preference for information to be presented concisely, with a brief description of the medicine, benefits, dosage, contraindications/cautions and a fully referenced research summary. The majority stated that they did not have time to digest large amounts of information and strongly preferred a summary.

### Personal and family use of CMs

Of the sample (n=12), nine respondents regularly took a CM (usually daily). In Group 1, two out of three took a CM, and none reported a family background of CM use. In Group 2, three out of five took CMs and only one had a family background of CM use (Traditional Chinese Medicines). They said that this probably had some influence on their openness to CM. In Group 3, all four respondents used CMs. Two had some family background, where the family had used multivitamins or other basic vitamin preparations, but none said that influenced their current recommendation patterns. One Group 3 member stated their interest had come about because in the past, family members had achieved no benefits from orthodox medicines, so they had looked elsewhere to find answers.

### Relationship with other healthcare professionals

Most respondents described a somewhat uncomfortable relationship with doctors. Only one pharmacist reported a very positive relationship. This respondent had worked in a pharmacy adjacent to a surgery, where doctors commonly recommended CMs, and this had caused the pharmacist to become more interested in the benefits of CMs. Several pharmacists in Groups 2 and 3 said some doctors were not happy with them recommending CMs to their patients and recalled instances where a doctor had told their patients not to take them. This was of concern to the pharmacists and caused tension between the two professions:

‘That can be frustrating, and subconsciously it can form a bit of mistrust between myself and the customer; when I’ve recommended something and the doctor has said, “don’t take it”. I worry about that sometimes.’

Communication between doctors and pharmacists was regarded as poor, and several respondents thought that doctors should become better educated about CMs for improved patient care.

‘I think if they had a broader understanding, that would benefit everyone.’

Many of the respondents had worked alongside a naturopath – all pharmacists in Group 3, one in Group 2 and one in Group 1. Those who had were all very positive about the value of having a naturopath in the pharmacy. They consistently mentioned referring customers to the naturopath, if they did not feel adequately knowledgeable, and learning from them about efficacy, safety and interactions relating to CMs:

‘The naturopath can keep right up to date and the pharmacist can feed off that and learn from the naturopath.’

## Discussion

Previous research investigating the integration of CMs into pharmacy practice has not examined in detail the factors that impact the decision of a pharmacist to recommend a CM product to a customer. This study has identified a range of influential factors, including pharmacy protocols relating to companion selling of CMs; the desire to provide health benefits to a customer; the pharmacist’s knowledge about CMs, which is linked to confidence in recommending based on efficacy and safety; awareness of evidence in support of CMs; the presence of a naturopath in the store (an increasingly common phenomenon in Australian pharmacies [26]); customer demand and positive feedback from customers; and the quality of products available and profile of the company providing them (Table 2).

Previous studies have reported the primary reason for patients choosing or being recommended a CM product is for particular health benefits – the maintenance of general health, prevention of disease and the treatment of minor conditions [7,8,13]. This correlates with the findings of this study, that patient benefit from CMs was a major motivating factor for pharmacists in all groups.

The concept of providing a health benefit differed somewhat between Group 2 and 3 respondents. Those in Group 2 were more likely to accept that a CM could offer health benefits if they were satisfied with the evidence provided, whether by a company or by independent sources, or if they had witnessed the benefit themselves. This concurs with previous studies that have found that knowledge of a particular CM and a proven mechanism of action are influential factors [6,7]. In contrast, Group 3 respondents (those who recommended CMs most frequently) were passionate about a ‘holistic approach’ and commonly linked health benefits with a ‘healthcare solution’ comprising not only a specific treatment, but also the provision of supportive measures to prevent recurrence and thereby improve patient care. This in turn was seen to be linked to business benefits. All Group 3 respondents were owners or managers of their stores, which may have influenced their emphasis on business-related benefits. Previous research has reported that profit is not an important motive for pharmacists [6,7,15], although there is an understanding that CM products are an important contributor to the business of pharmacy [16].

The provision of a ‘healthcare solution’ or adopting a ‘holistic approach’ was mentioned by the majority of Group 2 and 3 respondents. For most, this involved the practice of recommending a CM as a companion product for a pharmaceutical medicine, or recommending several products with a view to providing the best patient care. This professional behaviour of pharmacists has only recently been described. A 2010 Australian survey investigating the integration of CMs into pharmacy practice found that nearly half of the respondents described their practice as providing ‘integrative care’, which was defined as ‘recommending CMs together with conventional medicines as part of standard practice’ [16].

In banner pharmacies, the implementation of protocols for companion recommendation appeared to be a major driver of the recommendation of CMs. The four Group 2 respondents who worked in banner pharmacies all mentioned corporate companion selling protocols as a major driver. Group 3 respondents had all been instrumental in implementing such protocols in their stores; they embraced this approach in order to provide good customer service, and said it provided them with a feeling of job satisfaction. The role of companion selling protocols as a driver of recommendation of CMs by pharmacists has not previously been described. The existence of such protocols may cause pharmacists to feel compelled to learn more about CMs and in turn result in increased recommendation of CMs. It also raises the question to what extent the professional judgment of the pharmacist may be influenced by a protocol when deciding whether or not to recommended CMs to a customer.

Questions of whether the relationship with other healthcare professionals affected the recommendation of CMs provided some interesting insights. All but one respondent articulated either a neutral (n=4) or strained (n=4) relationship with doctors. The main contentious issue, identified by four respondents, was doctors dissuading patients from taking a CM that the pharmacist had recommended. The pharmacists were unhappy with this situation and claimed it was a difficult situation to deal with, as the communication between the two professions was poor.

The relationship with naturopaths was described as far more harmonious. Naturopaths were seen as being able to provide good quality information and were considered to be a valuable resource in the pharmacy. Five respondents worked alongside a naturopath, and one of these stated that because a naturopath was present, they would refer customers to the naturopath for a CM recommendation, rather than make it themselves. Three Group 3 respondents, who were store managers and had employed naturopaths, felt that the presence of a naturopath had not changed their recommendation behaviour, although they would often refer to the naturopath for more information about a CM. A previous Australian survey found that pharmacists who work alongside naturopaths find the service provided by naturopaths valuable, while the presence of a naturopath made pharmacists less likely to recommend CMs themselves [16].

Barriers to the recommendation of CMs were relatively clear-cut, with safety concerns (drug interactions and adverse effects) being the most important, identified by all respondents. Other identified barriers varied between groups. Group 1 felt inadequately trained. Group 2 reported a lack of evidence as a significant factor and felt the need to be confident in the patient benefit before recommending a CM. Group 3 respondents were the only to mention a lack of time as a reason for not recommending CMs. This may be because they also wanted to offer a more comprehensive ‘healthcare solution’, which no doubt takes some time to provide. Previous studies have identified a lack of knowledge or lack of evidence as the main barriers to recommending CM products [4,6,11,16-20,27]. As many pharmacists by their own admission do not have adequate training in CM, it is unclear whether in some cases they have truly assessed the evidence for a CM before reaching the conclusion that there is a lack of it.

All groups expressed discomfort with ‘fad’ products, and respondents claimed they would not recommend them if asked their opinion, while maintaining that customers have a right to choose such products, and thus they should be available from the pharmacy. Conversely, respondents considered some CM companies to be trustworthy and providing good quality and properly researched CM products. The trusted companies were considered to be the larger, more established ones with larger products ranges. Previous research has also found that poor product quality is a disincentive to recommend CMs [16].

There was an impetus to learn more about CMs, especially in Groups 2 and 3, which is encouraging, as the majority of Australian pharmacy customers expect the pharmacist to be able to provide accurate CM information [5]. Respondents consistently reported that customers are becoming more educated, are interested in preventative approaches and want to take more control of their healthcare, a picture also reported from Canada in the context of CM customers in pharmacies [28]. Therefore, significant motivating factors for Group 2 respondents to increase knowledge about CMs were likely the encouragement to follow pharmacy protocols for companion selling, customer demand and positive customer feedback.

The respondents seemed moderately satisfied with the CM educational materials available but did find room for improvement, both in terms of quality and presentation of the information. They preferred brief but professionally produced information that reflects similar resources for pharmaceuticals, including easily accessible online databases, which is consistent with the suggestion that evidence-based CM information should be included in professional handbooks and guidelines for pharmacists [16]. Interestingly, respondents in this study were also happy to get information from CM companies, as long as it is professionally presented with a clear description of levels of evidence to support efficacy.

A Canadian study has highlighted the professional and ethical dilemma faced by pharmacists who sell CMs without possessing sufficient knowledge about these products [29], and most respondents in the present study saw a clear need to incorporate more CM education into formal pharmacy training. Whilst there are some moves to do so [30,31], there seems to be ample evidence from this and other studies that the majority of pharmacists would like more CM training [4,6,8,16,27,32,33].

### Limitations

As with all studies relying on volunteer subjects, some degree of selection bias is likely, and our small sample is therefore unlikely to be truly representative of the population of community pharmacists in Australia. In particular, it may have been the case that pharmacists with a greater interest in CM would have been more likely to agree to take part in the study.

Another source of potential bias is that the primary investigator (SEC), who conducted the interviews, is an employee of a CM company, a fact the respondents were aware of. This dual role of the investigator could potentially have biased responses in favour of CM, but the desire of study subjects to please the investigators is always a potential source of bias, and we do not believe that this would have influenced responses in a significant way.

Although the small sample size and the above caveats clearly limit the generalisability of the findings, it is noteworthy that data saturation was reached, with no new themes emerging in the final interviews. This, combined with the in-depth nature of the interviews, allows for this study to make a valuable contribution to the understanding of the evolving interface between complementary medicine and pharmacy, in particular in Australia. It should also provide a sound basis for a larger qualitative study in the area.

## Conclusions

This study has explored the factors that affect Australian community pharmacists’ behaviour in terms of recommending CMs to customers. These factors are clearly many and diverse, and pharmacists’ knowledge of, confidence with and inclination to recommend CMs vary. We have for the first time described protocols for companion selling of CMs as an important driver of the recommendation of CMs, especially for pharmacists working in banner stores. This is an important finding, because it suggests that corporate pharmacy groups currently play a significant role in how many Australian pharmacists recommend CMs to their customers.

Due to the widespread use of CMs by the Australian public and the fact that many of these products are sold in pharmacies, improving education for pharmacists in this area is important. In line with many previous studies, we found that most pharmacists feel ill prepared to provide the public with adequate advice about the many CM products in their stores. Improving CM education ought to be a priority for all providers of pharmacy training, and continuing professional education materials and other information resources, whether produced by industry or independent agencies, must be rigorous, of high standard and easy for pharmacists to access.

## Abbreviation

CM(s): Complementary medicine(s).

## Competing interests

SEC is an employee of the Australian natural medicine company Blackmores Ltd., which provided her with an educational grant and access to customer information for the purpose of the study. The company had no involvement in the study design and execution, data analysis and interpretation, or the preparation of the manuscript. HW declares that he has no competing interests.

## Authors contributions

SEC designed and carried out the research and drafted the manuscript as part of her Master of Clinical Science degree. HW supervised the project and contributed to study design, data analysis and manuscript preparation. Both authors read and approved the final manuscript.

## Pre-publication history

The pre-publication history for this paper can be accessed here:

http://www.biomedcentral.com/1472-6882/12/183/prepub
